# Coordinated Role of Toll-Like Receptor-3 and Retinoic Acid-Inducible Gene-I in the Innate Response of Bovine Endometrial Cells to Virus

**DOI:** 10.3389/fimmu.2017.00996

**Published:** 2017-08-23

**Authors:** Luisa C. Carneiro, Carmen Bedford, Sarah Jacca, Alfonso Rosamilia, Vera F. de Lima, Gaetano Donofrio, I. Martin Sheldon, James G. Cronin

**Affiliations:** ^1^Institute of Life Science, Swansea University Medical School, Swansea, United Kingdom; ^2^Faculty of Agricultural and Veterinary Science, Universidade Estadual Paulista, Jaboticabal, Brazil; ^3^Department of Medical-Veterinary Science, University of Parma, Parma, Italy

**Keywords:** endometrium, pattern-recognition receptor, toll-like receptor, virus, cytokine, chemokine, poly(I:C), RIG-I

## Abstract

Bovine herpesvirus-4 (BoHV-4) and bovine viral diarrhea virus (BVDV) infect the uterus of cattle, often resulting in reduced fertility, or abortion of the fetus, respectively. Here, exposure of primary bovine endometrial cells to BoHV-4 or BVDV modulated the production of inflammatory mediators. Viral pathogen-associated molecular patterns (PAMPs) are detected *via* pattern-recognition receptors (PRRs). However, the relative contribution of specific PRRs to innate immunity, during viral infection of the uterus, is unclear. Endometrial epithelial and stromal cells constitutively express the PRR Toll-like receptor (TLR)-3, but, the status of retinoic acid-inducible gene I (RIG-I), a sensor of cytosolic nucleic acids, is unknown. Primary endometrial epithelial and stromal cells had low expression of RIG-I, which was increased in stromal cells after 12 h transfection with the TLR3 ligand Poly(I:C), a synthetic analog of double-stranded RNA. Furthermore, short interfering RNA targeting *TLR3*, or interferon (IFN) regulatory transcription factor 3, an inducer of type I IFN transcription, reduced Poly(I:C)-induced RIG-I protein expression and reduced inflammatory mediator secretion from stromal cells. We conclude that antiviral defense of endometrial stromal cells requires coordinated recognition of PAMPs, initially *via* TLR3 and later *via* inducible RIG-I.

## Introduction

The initiation of the innate immune response to viruses depends on the detection of pathogen-associated molecular patterns (PAMPs) by pattern-recognition receptors (PRRs) (1). The main families of PRRs are the Toll-like receptors (TLRs), nucleotide oligomerization domain-like receptors, retinoic acid-inducible gene I (RIG-I)-like receptors, and C-type lectin receptors. These receptors are most often expressed by hematopoietic cells, but the epithelial and stromal cells of the endometrium also possess functional PRRs. The PRRs that detect uterine bacteria that contaminate the uterus, causing infertility, are well documented in cattle (2–9). However, viral infections also result in decreased conception rates and abortion in cattle. Although, little is known about the functional response of endometrial cells in the detection and response to viruses or their PAMPs.

The double-stranded (ds)DNA *Gammaherpesvirus* bovine herpesvirus-4 (BoHV-4) and the single-stranded (ss)RNA *Pestivirus* bovine viral diarrhea virus (BVDV) infect the uterus of cattle (10, 11). *Gammaherpesviruses* are ubiquitous pathogens of animals and humans, and primary infections are usually subclinical. However, BoHV-4 and BVDV can cause disease, particularly if the host becomes immunologically or metabolically stressed, a common occurrence in postpartum dairy cattle (12). BoHV-4 is tropic for endometrial epithelial and stromal cells, and macrophages become persistently infected with virus (13). Increased replication of BoHV-4 also occurs in the endometrium particularly following postpartum infection of the uterus with the Gram-negative *Escherichia coli*, resulting in uterine disease (14). Lipopolysaccharide (LPS), from the Gram-negative cell wall, induces inflammatory cytokine secretion from endometrial cells *via* TLR4 (3, 15). Another commonly isolated virus BVDV can result in abortion of the fetus or in the birth of a persistently infected calf, depending on the stage of gestation, and the development of the fetal immune system (10).

The innate immune system is the first line of resistance to viral infections, and plays a pivotal role in both the host’s early response to viruses and subsequent adaptive immunity (16, 17). During viral infections, endosomal TLRs, and cytoplasmic RNA helicases, including RIG-I, detect PAMPs, such as nucleic acids, and initiate antiviral immunity *via* the induction of inflammatory mediators and interferons (IFNs) (1). DNA viruses are usually sensed *via* the DNA-dependent activator of interferon regulatory factor (IRF) (DAI) that senses B-form DNA and induces type I IFNs (18). However, DAI is not present in all cell types and studies in DAI-deficient mice have failed to identify essential roles for DAI in an innate antiviral response to herpesviruses (19). Therefore, DAI is not thought to be the main PRR in innate defense against herpesviruses. Viral DNA is also sensed by cyclic GMP-AMP synthase (cGAS) and gamma-interferon-inducible protein, which depend on the adaptor protein stimulator of IFN genes (STING) (20, 21). However, DNA viruses can also be sensed indirectly by the endosomal TLR3 after translation of dsRNA during viral replication (22). dsRNA is part of the life-cycle of all viruses, except negative-stranded RNA viruses (23). Activation of TLR3, through binding of dsRNA, utilizes a MyD88-independent pathway, *via* the TIR-domain-containing adapter-inducing IFN-β (TRIF). Activation of this pathway leads to phosphorylation of nuclear-factor κB (NF-κB), which stimulates inflammatory cytokine gene expression. Alternatively, TRIF signaling can activate TANK binding-kinase-1 (TBK-1) and IRF-3, which is a key transcription factor responsible for type I IFN gene expression (24, 25).

Viral DNA is also transcribed to uncapped 5’-triphosphate ssRNA by RNA polymerase III, which is sensed by cytosolic RIG-I (16, 26–28). *Pestivirus*, such as BVDV, and *Hepacivirus* are the only mammalian viruses that include an uncapped 5’-triphosphate ssRNA stage in their replication cycles (26, 29). Dendritic cells (DCs) recognize Herpes simplex virus-2 *via* TLR9 (30). However, studies have also shown a direct interaction of herpesvirus DNA and RIG-I, indicating a non-redundant role for the sensing of herpesvirus by fibroblasts (31, 32). Furthermore, BVDV induces expression of *TLR3* and *DDX58* (RIG-I) genes in bovine endometrial cells (8).

In the present study, we investigated the innate immune response of the endometrium to BVDV, BoHV-4, and to representative PAMPs of the virus life cycle. In order to understand the recognition of viruses by endometrial epithelial and stromal cells and the induction of pro-inflammatory mediators and IFNs, we investigated the role of TLR3 and RIG-I in the innate immune response to viable BoHV-4 and BVDV, and viral PAMPs. We show that endometrial stromal cells responded to viable viruses by inducing inflammatory cytokines and chemokines. However, epithelial and stromal cells did not directly respond to transfected DNA or 5’-triphosphorylated ssRNA, but initiate an innate immune response to dsRNA, which was TLR3 dependent. Although, unchallenged stromal cells did not express RIG-I. RIG-I levels increased following dsRNA Poly(I:C) transfection, and this was dependent on TLR3. Therefore, the endometrial innate immune response required a co-ordinated response to virus, involving TLR3 mediated pro-inflammatory cytokine and chemokine expression, and dsRNA induced RIG-I expression. Understanding the mechanisms of innate immune activation of endometrial cells, and the etiology and pathogenesis of disease, may allow the development of efficient antiviral strategies.

## Materials and Methods

### Endometrial Cell Culture

Uteri with no gross evidence of genital disease or microbial infection were collected from cattle processed as part of the normal work of an abattoir, as described previously (3). Endometrial epithelial and stromal cell populations were isolated, and the absence of immune cell contamination confirmed by cell morphology and by FACS analysis, as described previously (2). The cells were plated at a density of 1 × 10^5^ cells/ml in 6-, 12- or 24-well plates (TPP, Trasadingen, Switzerland) in complete medium: RPMI 1640 (Gibco, Life Technologies, UK), supplemented with 10% heat-inactivated, endotoxin-free, fetal bovine serum (BioSera, East Sussex, UK), 50 IU/ml penicillin (Sigma-Aldrich Ltd., Dorset, UK), 50 µg/ml streptomycin (Sigma-Aldrich Ltd.), 2.5 µg/ml amphotericin B (Sigma-Aldrich Ltd.), and maintained in a humidified, 5% CO_2_ in air atmosphere incubator at 37°C.

Endometrial tissue for organ culture was collected from the intercaruncular areas of the endometrium, using sterile 8-mm diameter biopsy punches (Stiefel Laboratories Ltd., High Wycome, UK), as described previously (33). Tissues were cultured in 24-well plates (TPP, Trasadingen, Switzerland) containing 2 ml complete medium/well. The *ex vivo* organ cultures (EVOCs) were exposed to LPS (1 µg/ml), viable BoHV-4 (1 × 10^6^/ml virus particles) or BVDV (1 × 10^6^/ml virus particles) within 4 h of slaughter and maintained in a humidified, 5% CO_2_ in air atmosphere incubator at 37°C. Subsequently, supernatants were collected after 24 or 72 h for analysis of inflammatory mediators by enzyme-linked immunosorbent assay (ELISA).

### Monocyte-Derived DCs

Forty-five milliliters of bovine blood, collected from the *vena jugularis externa*, were immediately transferred into a tube containing 5 ml sodium citrate (3.2%; Sigma-Aldrich) at the local abattoir. In the laboratory, the blood was mixed with an equal volume of Dulbecco’s phosphate buffered saline (D-PBS; Sigma-Aldrich) and 16 ml of this diluted blood sample was carefully layered onto 12 ml Ficoll-Paque PLUS (1.084 g/ml; GE Healthcare, UK) in a 50 ml centrifuge tube (Falcon, UK), which was centrifuged for 40 min at 400 × *g*. The mononuclear layer was carefully collected from the interface, between the red-blood cells and lymphocytes, and transferred to a sterile 15 ml centrifuge tube (Falcon, UK) and D-PBS was added to the tube to a final volume of 14 ml. The cells were resuspended by gentle pipetting and the tube was centrifuged at 400 × *g* for 15 min. The supernatant was removed and the mononuclear cells resuspended in 14 ml of D-PBS. After centrifugation at 400 × *g* for 10 min, cells were resuspended in 3 ml serum-free RPMI 1640 and transferred to a 6 cm diameter Petri dish (NUNC, UK). After 30 min, the cells were washed in 6 ml D-PBS and cultured in 3 ml complete medium supplemented with IL-4 (25 ng/ml; Kingfisher Biotech, St. Paul, MN, USA) and GM-CSF (25 ng/ml; Kingfisher Biotech) to induce differentiation into DCs. Media was half-changed every 3 days, to avoid disturbing the cells, and the cells were cultured for 9 days before treatment.

### Endometrial Cell Culture Treatments

#### Viruses

Bovine herpesvirus-4-4EGFPΔTK and the NADL strain of BVDV (34) were propagated by infecting confluent monolayers of Madin–Darby bovine kidney cells (MDBK) at a multiplicity of infection (m.o.i.) of 0.5 tissue cell infectious doses/50 (TCID_50_) per cell and maintained in minimal essential media (MEM; Gibco, UK) with 2% FBS for 2 h. The medium was then removed and replaced with fresh MEM containing 10% FBS. The virus was purified when 90% of the cell monolayer exhibited a cytopathic effect (CPE), at approximately 72 h post-infection. Cell-associated virions were freed by three cycles of freeze-thawing at −80°C. Cell debris was removed by centrifugation, and virions were pelleted through a 3 ml cushion of 30% sucrose in PBS, in a Beckman 70 Ti rotor at 35,000 rpm for 90 min at 4°C. Viral pellets were resuspended in cold MEM without FBS and TCID_50_ were determined on MDBK cells by serial dilutions (35).

Bovine endometrial stromal or epithelial cells were challenged with BoHV-4 or BVDV at 1 m.o.i.. Explants were challenged with 1 × 10^6^/ml virus particles. The supernatants were harvested and analyzed by ELISA.

#### Pathogen-Associated Molecular Patterns

Ninety per cent confluent endometrial epithelial cells, stromal cells, or monocyte-derived DCs were challenged with the following PAMPs: dsDNA CpG dsDNA (ODN 2007), ssRNA (ssPolyU Naked), dsRNA Poly(I:C) low-molecular weight (LMW), dsRNA Poly(I:C) high-molecular weight (HMW), with Lipid A or ultrapure LPS from *E. coli* 0111:B4 as a positive control (all InvivoGen, Toulouse, France) for the times and concentrations indicated in Section “Results.” Prior to challenge, PAMPs were diluted in OPTI-MEM media (Gibco, UK), containing DOTAP (*N*-[1-(2,3-Dioleoyloxy)propyl]-*N*,*N*,*N*-trimethylammonium methyl-sulfate) liposomal transfection reagent (Sigma-Aldrich Ltd.), and incubated for 5 min at room temperature. The DOTAP/PAMP solution was then added to 1 ml of complete media in a well of a 24-well plate, so that the final concentration of DOTAP was 10 µg/ml. Each experiment was performed using cells isolated from at least three independent animals. Supernatants were collected and stored at −20°C, while cells were washed, and cell lysates collected using PhosphoSafe™ Extraction Reagent (EMD Millipore, UK) and stored at −80°C until further processing for immunoblotting.

#### Enzyme-Linked Immunosorbent Assay

Concentrations of bovine IL-6 or IL-1β were measured by ELISA according to the manufacturer’s instructions (Bovine IL-6 ELISA Reagent Kit ESS0029; Bovine IL-1 beta ELISA Reagent Kit ESS0027; Thermo Scientific, Cramlington, UK). Bovine IL-8 was measured by ELISA, as described previously (36).

#### Immunoblotting

Total cell lysate proteins were quantified using the DC Assay (Bio-Rad, UK) and separated (10 μg/lane) using Laemmli buffer (Sigma-Aldrich) and 10% (vol/vol) SDS-PAGE, as described previously (15). Pre-stained molecular weight markers were run in parallel lanes (Bio-Rad, UK). After electrophoresis, proteins were transferred to a polyvinylidene difloride membrane (GE Healthcare); non-specific sites were blocked using a solution of 5% (wt/vol) BSA (Sigma-Aldrich) in Tris-buffered saline (TBS; Sigma-Aldrich) for 1 h at 37°C with gentle agitation. Membranes were probed with antibodies targeting RIG-I (#4200, Cell Signaling, Danvers, MA, USA); phosphorylated p65 (Serine536; #3033, Cell Signaling); total p65 (#3987, Cell Signaling); and phosphorylated TBK1 (serine172; #5483, Cell Signaling). Protein loading was evaluated by examining β-actin protein levels using a β-actin antibody (ab8226, Abcam, UK). Primary antibodies were used at 1:1,000 dilutions in 5% (wt/vol) BSA, TBS, and 0.1% Tween 20 (pH 7.6; Sigma) overnight at 4°C with gentle agitation. After incubation, membranes were washed for 5× 5 min in TBS and 0.1% Tween 20. Membranes were then incubated in secondary horseradish peroxidase-conjugated antibody (Cell Signaling) in TBS and 0.1% Tween 20 for 1.5 h, and washed for 5× 5 min in TBS and 0.1% Tween 20 (pH 7.6). Steady-state levels of immunoreactive proteins were visualized using enhanced chemiluminescence (Western C, Bio-Rad). Densitometry of non-saturated signals was performed on independent immunoblots, using the Quantity-one software (Bio-Rad).

### Short Interfering RNA (siRNA)

Primary endometrial epithelial and stromal cells were transfected using Lipofectamine RNAiMAX Reagent (Invitrogen) and siRNA (designed using Dharmacon’s siDESIGN Center, Thermo Fisher Scientific, UK) targeting *TLR3, IRF3, TRAF3*, or *MYD88* (duplex sequences in Supplemental Table 1), as described previously (15). Briefly, RNAiMAX–RNAi duplex complexes were formed by adding 50 pM of siRNA to 500 µl of Opti-MEM I Reduced Serum Media (Invitrogen) in each well of a 6-well plate, with 50 pM of ON-TARGETplus Non-targeting siRNA #1 (Dharmacon) as a control. Then, 7.5 µl RNAiMAX was added to each well containing the diluted RNAi molecules and left for 20 min at room temperature. Exponentially growing cells (7 × 10^5^ epithelial cells, 5 × 10^5^ stromal cells) were then seeded in 2.5 ml of RPMI 1640 growth media, supplemented with 10% FBS, per well to give approximately 50% confluency. Poly(I:C) (1 µg/ml) challenge was carried out 48 h after siRNA treatment of cells, and cells and supernatants collected after a further 24 h, for immunoblotting and ELISA experiments. Viable BoHV-4 or BVDV challenge (1 m.o.i.) was carried out 48 h after siRNA treatment of cells, and cells and supernatants collected after a further 72 h, for immunoblotting and ELISA experiments.

### Statistical Analysis

Endometrial cells were isolated independently, with the animal designated as the statistical unit. Data are presented as mean + SEM and treatments were compared by two-way analysis of variance with Dunnett’s post-comparison test, unless otherwise stated in Section “Results,” using SPSS 16.0 (SPSS Inc.). Values of *P* < 0.05 were designated as significant.

## Results

**Figure 1:**
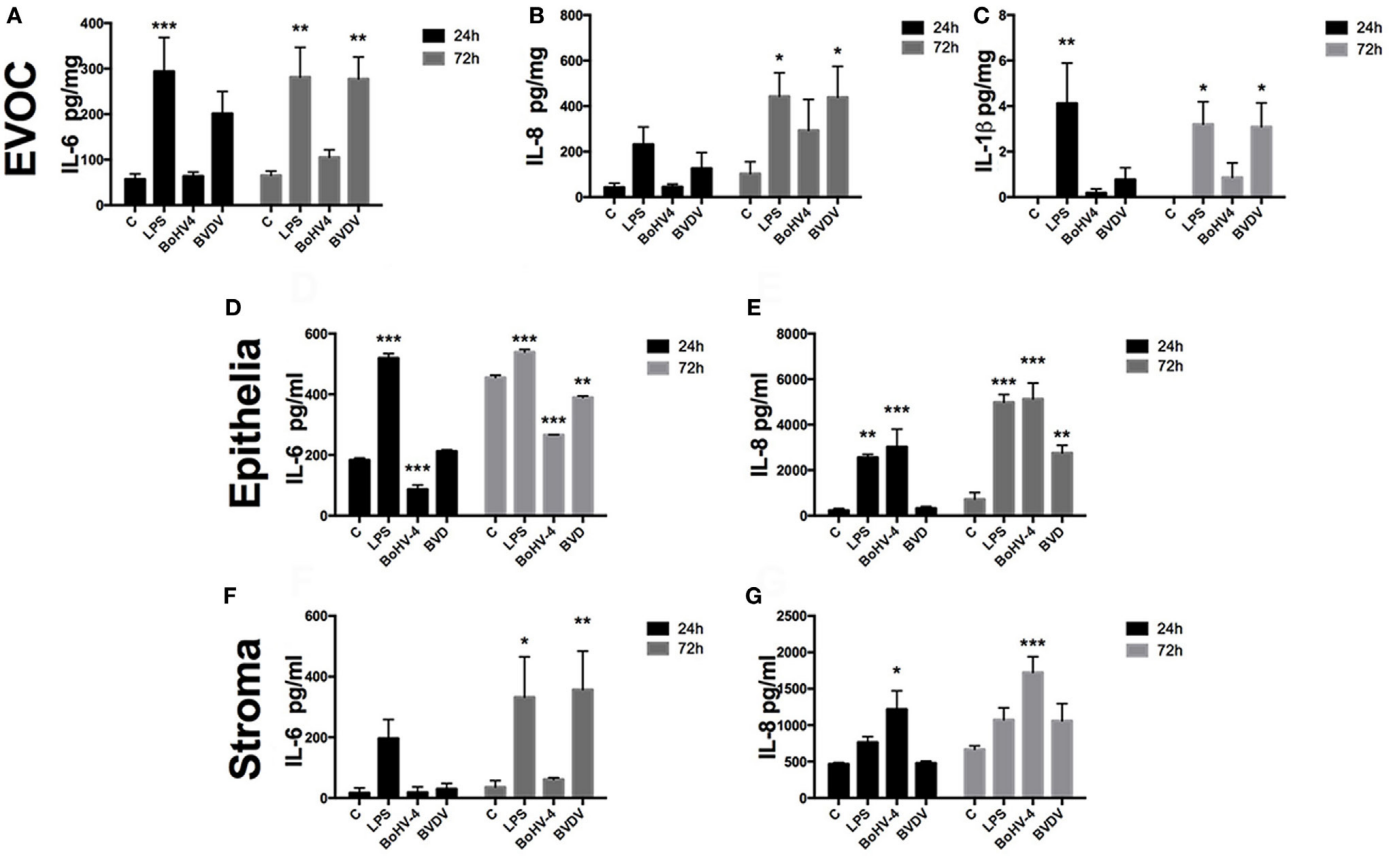
Viable bovine herpesvirus-4 (BoHV-4) and bovine viral diarrhea virus (BVDV) induce inflammatory responses in endometrial tissue and cells. *Ex vivo* organ cultures [EVOCs; **(A–C)**; *n* = 6 independent animals], epithelial cells [**(D,E)**; *n* = 4], or stromal cells [**(F,G)**; *n* = 4] were exposed to lipopolysaccharide (1 µg/ml), BoHV-4 [cells at 1 multiplicity of infection (m.o.i.); explants at 1 × 10^6^ viral particles/ml], BVDV (cells at 1 m.o.i.; explants at 1 × 10^6^ viral particles/ml) for 24 or 72 h. Concentrations of IL-6 **(A,D,F)**, IL-8 **(B,E,G)**, and IL-1β **(C)** in supernatants were measured by enzyme-linked immunosorbent assay. Data are presented as mean + SEM, and analyzed by analysis of variance, using the *post hoc* Dunnett’s multiple comparison test to compare treatments with control, ***P < 0.001, **P < 0.01, *P < 0.05.

To characterize the innate immune response of the endometrium to viruses, endometrial EVOCs, endometrial epithelial cells, and endometrial stromal cells were exposed to BoHV-4 or BVDV virus for 24 or 72 h. The prototypical PAMP, LPS, was used as a positive control, since LPS induces inflammatory cytokine secretion from endometrial cells (3, 15, 33). Indeed, EVOC supernatants accumulated IL-6, IL-8, and IL-1β (Figures 1A–C), and epithelial (Figures 1D,E) and stromal cells (Figures 1F,G) accumulated IL-6 and IL-8 in response to LPS.

Bovine herpesvirus-4 did not induce cytokine production in EVOCs, whereas BVDV induced production of IL-6, IL-8, and IL-1β (Figures 1A–C). BoHV-4 and BVDV resulted in reduced accumulation of IL-6, but increased IL-8 accumulation from epithelial cells, when compared with control (Figures 1D,E). Whereas, supernatants of stromal cells accumulated IL-6 after exposure to BVDV and IL-8 on exposure to BoHV-4 (Figures 1F,G). Taken together, these data provide evidence that endometrial tissue and cells sense and respond to viruses that commonly induce uterine disease, by modulating inflammatory mediator production. However, this response seems to depend on the virus and the cell type of the endometrium.

**Figure 2:**
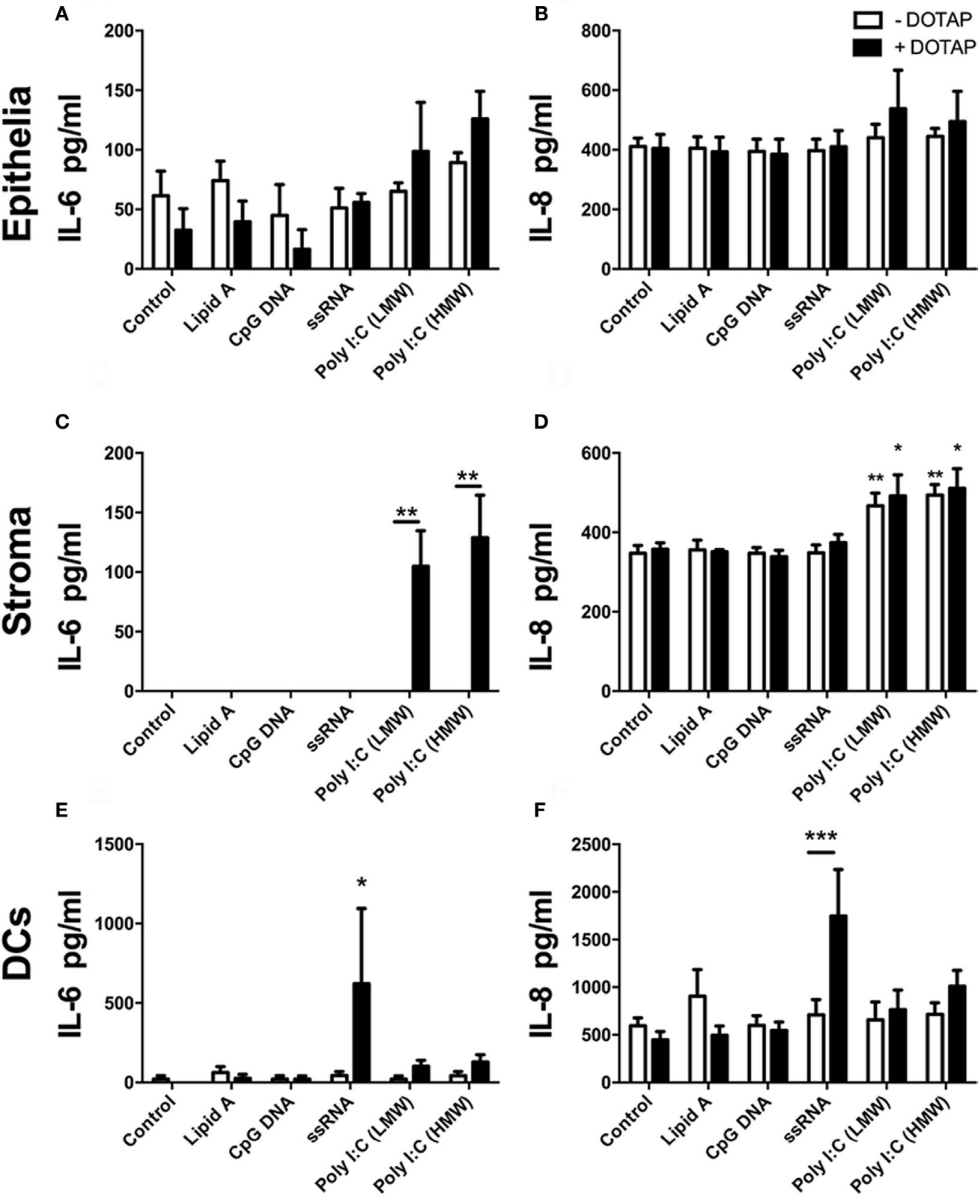
Double-stranded RNA (dsRNA) induces inflammatory mediator production in endometrial cells and dendritic cells (DCs). Epithelial cells [**(A,B)**; *n* = 7 independent animals], stromal cells [**(C,D)**; *n* = 5], or DCs [**(E,F)**; *n* = 4] were exposed to (white bars) or transfected (+DOTAP; black bars) with Lipid A (0.1 µg/ml), CpG DNA (1 µg/ml), ssRNA (1 µg/ml), dsRNA Poly(I:C) (low-molecular weight; 1 µg/ml), or Poly(I:C) (high-molecular weight; 1 µg/ml) for 24 h. Concentrations of IL-6 **(A,C,E)** and IL-8 **(B,D,F)** in supernatants were measured by enzyme-linked immunosorbent assay. Data are presented as mean + SEM, and analyzed by two-way analysis of variance, using the *post hoc* Dunnett’s multiple comparison test to compare treatment with control, ***P < 0.001, **P < 0.01, *P < 0.05.

Next, we explored whether transfection of PAMPs, analogous to those produced at different stages of virus life-cycle, induced production of cytokines, or chemokines from endometrial epithelial and stromal cells. BoHV-4 is a dsDNA virus, and TLR9 detects unmethylated CpG dinucleotides in viral DNA, inducing innate immune responses *via* the adaptor MyD88 (30, 37). Here, transfected unmethylated CpG dsDNA (ODN 2007) did not significantly induce accumulation of IL-6 (Figures 2A,C) or IL-8 (Figures 2B,D) from endometrial epithelial or stromal cells. Uterine DCs have been implicated in pregnancy maintenance and serve as antigen-presenting cells with the ability to induce primary immune responses (38). DCs recognize Herpes simplex virus-2 *via* TLR9 (30). So, next we investigated the response of blood-derived DCs to transfected unmethylated CpG dsDNA (ODN 2007). DC supernatants did not accumulate IL-6 or IL-8 in response to transfected ODN 2007 (Figures 2E,F).

Bovine viral diarrhea virus is a positive ssRNA virus. Endosomal TLR7 and TLR8 recognize ssRNA (39, 40). Here, transfected ssRNA did not induce increased IL-6 or IL-8 in epithelial or stromal cell supernatants (Figures 2A–D). However, DCs did accumulate IL-6 and IL-8 in response to transfected ssRNA (Figures 2E,F).

**Figure 3:**
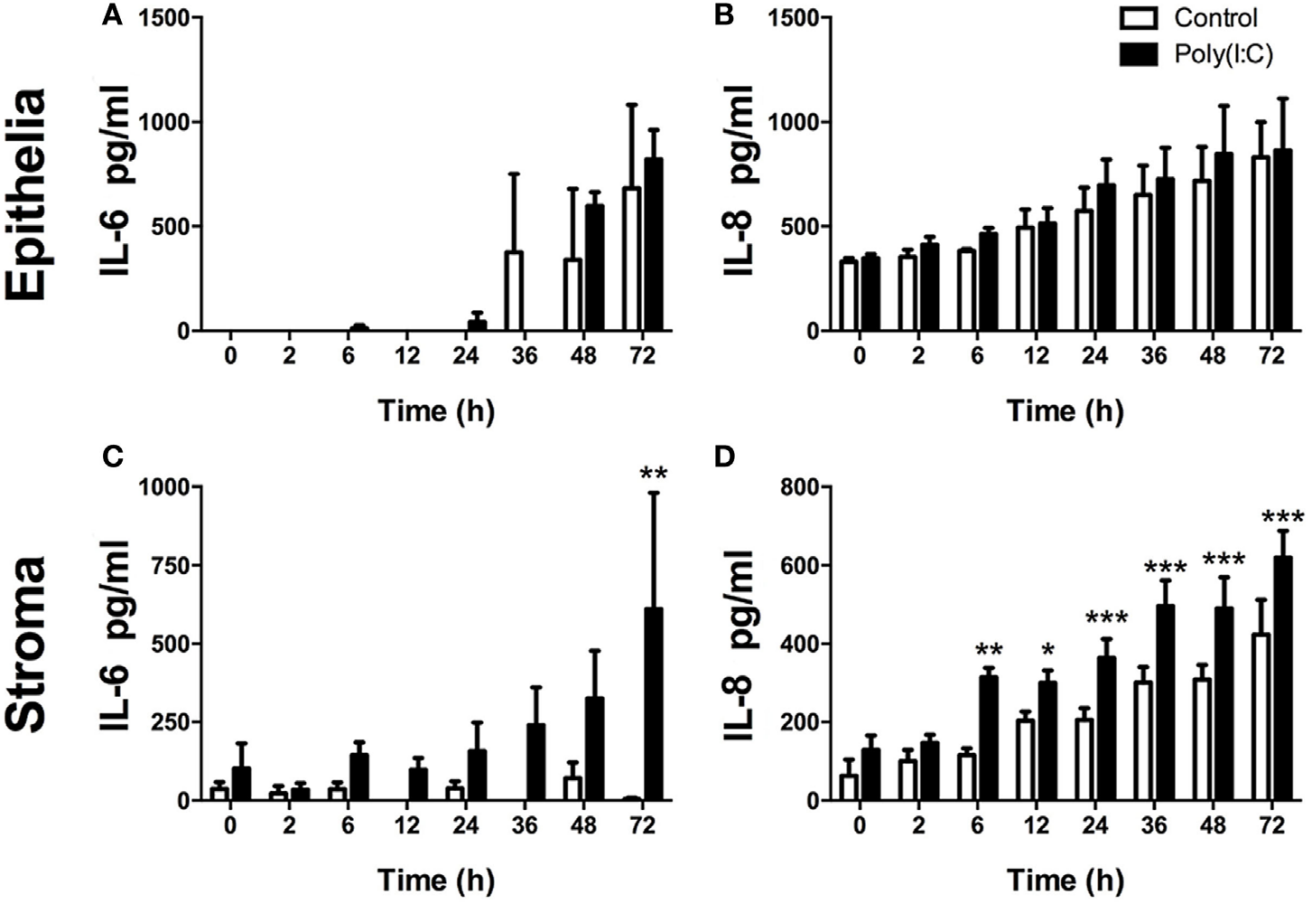
Double-stranded RNA (dsRNA) induces inflammatory mediator production in endometrial cells in a time-dependent manner. Epithelial [**(A,B)**; *n* = 3 independent animals] or stromal cells [**(C,D)**; *n* = 4] were transfected with dsRNA Poly(I:C) (1 µg/ml) for 2–72 h **(A–D)**. Concentrations of IL-6 **(A,C)** or IL-8 **(B,D)** in supernatants were measured by enzyme-linked immunosorbent assay. Data are presented as mean + SEM, and analyzed by two-way analysis of variance, using the *post hoc* Dunnett’s multiple comparison test to compare treatment to control at each time-point, ***P < 0.001, **P < 0.01, *P < 0.05.

As a PAMP, dsRNA is an important activator of innate immunity against viral infection (1). Here, Poly(I:C), which mimics dsRNA, did not induce increased accumulation of IL-6 or IL-8 from epithelial cells or DCs (Figures 2A,B,E,F). Whereas, Poly(I:C) induced IL-6 and IL-8 production from stromal cells (Figures 2C,D). With stromal cells, Poly(I:C) did not induce IL-6 accumulation in cell supernatants without the transfection reagent DOTAP. This indicates that the induction of IL-6 by Poly(I:C) requires initiation of intracellular sensing pathways (Figure 2C). Furthermore, Poly(I:C) induced a time-dependent increase in IL-6 and IL-8 accumulation from stromal cells, but not epithelial cells (Figures 3A–D). This indicates that Poly(I:C) activates pathways that lead to proinflammatory production in stromal cells, but not epithelial or DCs.

**Figure 4:**
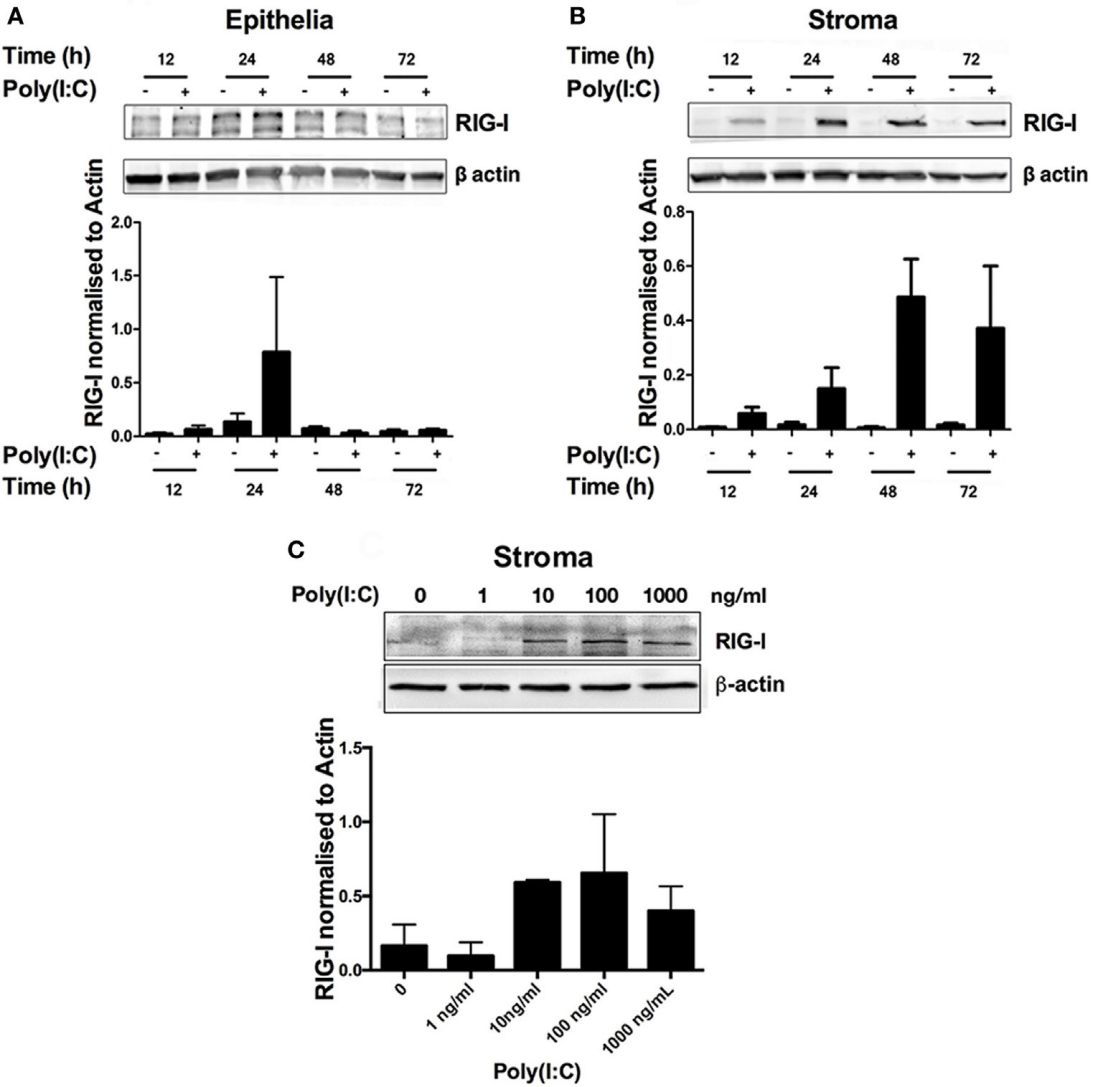
Double-stranded RNA (dsRNA) induces retinoic acid-inducible gene I (RIG-I) expression in endometrial stromal cells in a time- and concentration-dependent manner. Epithelial **(A)** or stromal cells **(B)** were transfected with dsRNA Poly(I:C) (1 µg/ml) for 2–72 h **(A,B)** or in increasing concentrations for 24 h **(C)**. Total cell proteins were extracted, and analyzed by immunoblotting for RIG-I. Average peak densities of RIG-I were normalized to β-actin and are presented as mean + SEM. Immunoblots are representative of two independent animal experiments.

Poly(I:C) binds to and triggers the activation of the RNA sensors endosomal TLR3 and RIG-I (also known as DDX58), among others (1). Bovine endometrial cells are known to express *TLR3* and *DDX58* genes (5, 8). Thus, we investigated RIG-I status in bovine endometrial cells in response to Poly(I:C). RIG-I was barely detectable in untreated cells (Figures 4A,B), but RIG-I was induced in a time- and concentration-dependent manner in endometrial stromal cells (Figures 4B,C).

**Figure 5:**
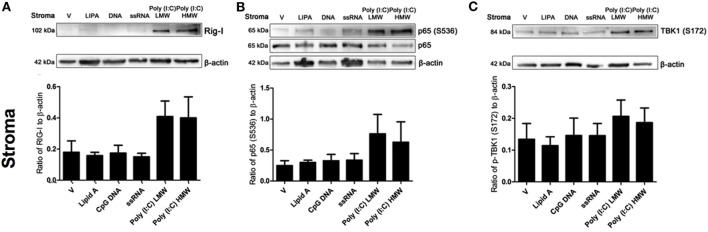
Double-stranded RNA (dsRNA) induces retinoic acid-inducible gene I (RIG-I) expression and phosphorylation of p65 and TANK binding-kinase-1 (TBK1) in endometrial stromal cells. Stromal cells **(A,B,C)** were transfected with Lipid A (0.1 µg/ml), CpG DNA (1 µg/ml), ssRNA (1 µg/ml), dsRNA Poly(I:C) (low-molecular weight; 1 µg/ml), or Poly(I:C) (high-molecular weight; 1 µg/ml) for 24 h. Total cell proteins were extracted, and analyzed by immunoblotting for RIG-I **(A)**, p65 or phosphorylated p65 (S536) **(B)**, and phosphorylated TBK1 (S172) **(C)**. Average peak densities of RIG-I, phosphorylated p65, or phosphorylated TBK1 proteins were normalized to β-actin and are presented as mean + SEM. Immunoblots are representative of two independent animal experiments.

The activation dynamics of TLR3 by Poly(I:C) are influenced by various factors, including size of the ligands. Therefore, we tested the effects of transfecting LMW or HMW Poly(I:C) on key innate immune signaling molecules initiated by viral infections, using the previously tested PAMPs as comparators. Neither DNA or ssRNA induced RIG-I expression, or phosphorylation of NF-kB (serine536) or TBK1 (serine172), key innate immune signaling intermediates in antiviral immunity (Figures 5A–C). Whereas, both transfected LMW and HMW Poly(I:C) induced expression of RIG-I, phosphorylation of NF-kB (serine536), and phosphorylation of TBK1 (serine172) in endometrial stromal cells (Figures 5A–C).

**Figure 6:**
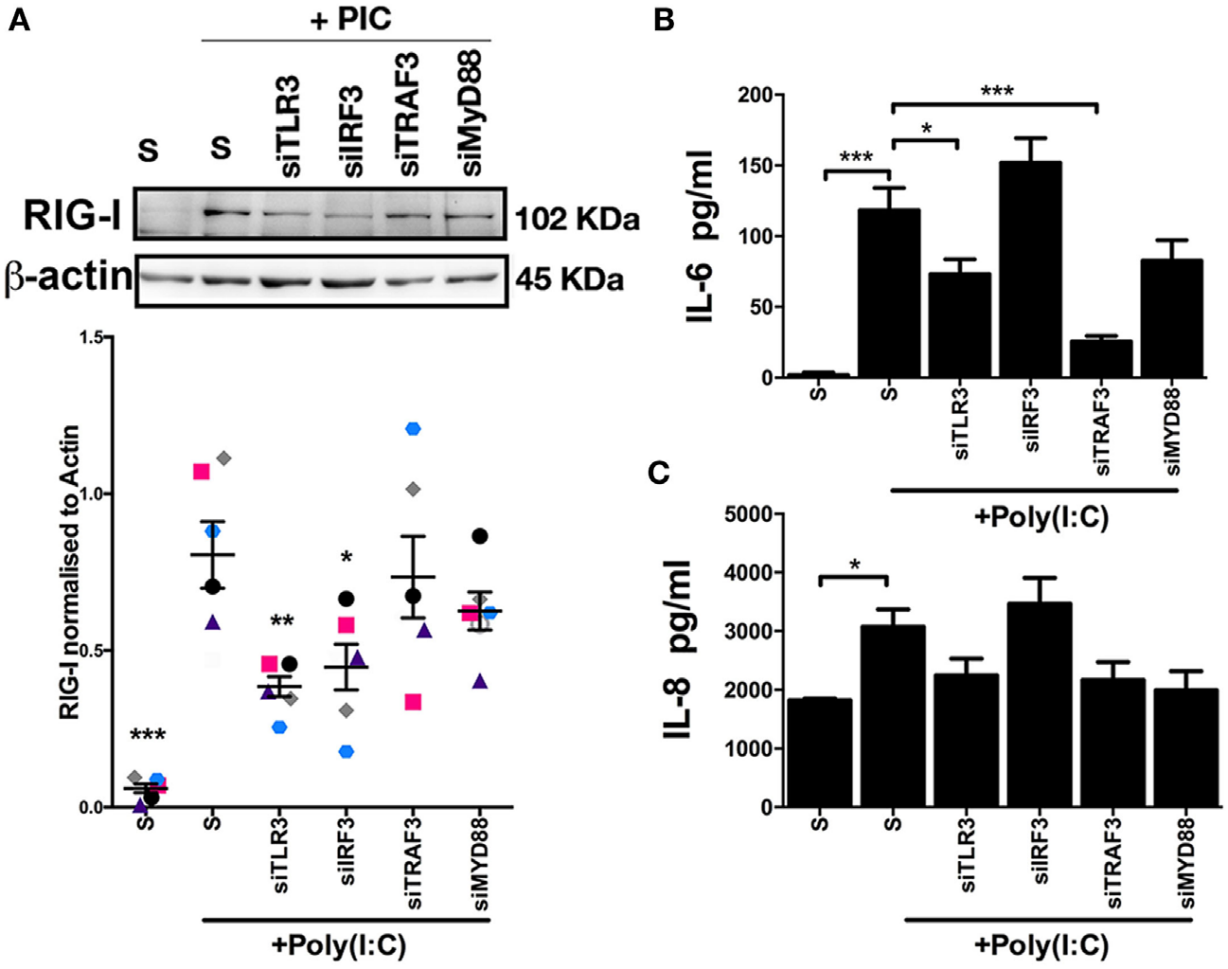
Inflammatory mediators moderate double-stranded RNA (dsRNA)-dependent retinoic acid-inducible gene I expression, and IL-6 and IL-8 production in endometrial stromal cells. Stromal cells **(A,B,C)** were cultured for 24 h in medium plus scrambled short interfering RNA (siRNA) (S) or media containing dsRNA Poly(I:C) (1 µg/ml). In each independent set of experiments, cells received vehicle plus scrambled siRNA control (S), vehicle plus siRNA targeting Toll-like receptor-3, interferon regulatory factor 3, TRAF3, or MYD88 18 h before 24 h transfected Poly(I:C) treatment. Concentrations of IL-6 **(B)** or IL-8 **(C)** in supernatants were measured by enzyme-linked immunosorbent assay. Data represent five independent animal experiments. Data are presented as mean + SEM, and analyzed by two-way analysis of variance, using the *post hoc* Dunnett’s multiple comparison test to compare siRNA plus Poly(I:C) to S plus Poly(I:C), ***P < 0.001, **P < 0.01, *P < 0.05.

As NF-kB and TBK1 signal downstream of activated TLR3 (24, 25), we next investigated TLR3 and related signaling molecules for their influence on RIG-I expression, and IL-6 and IL-8 accumulation in endometrial stromal cell supernatants. Depletion of *TLR3*, using siRNA, resulted in reduced RIG-I protein expression in Poly(I:C) transfected stromal cells (Figure 6A). Furthermore, depletion of *IRF3*, a signaling molecule downstream of TLR3, resulted in reduced RIG-I protein expression (Figure 6A). Whereas, depletion of *TLR3* or *TRAF3* resulted in reduced IL-6 accumulation in Poly(I:C) transfected stromal cells (Figures 6B,C). This indicates that TLR3 and IRF3 are important signaling pathways for RIG-I expression. However, TRAF3 is important for IL-6 production in stromal cells, but is not involved in the expression of RIG-I.

**Figure 7:**
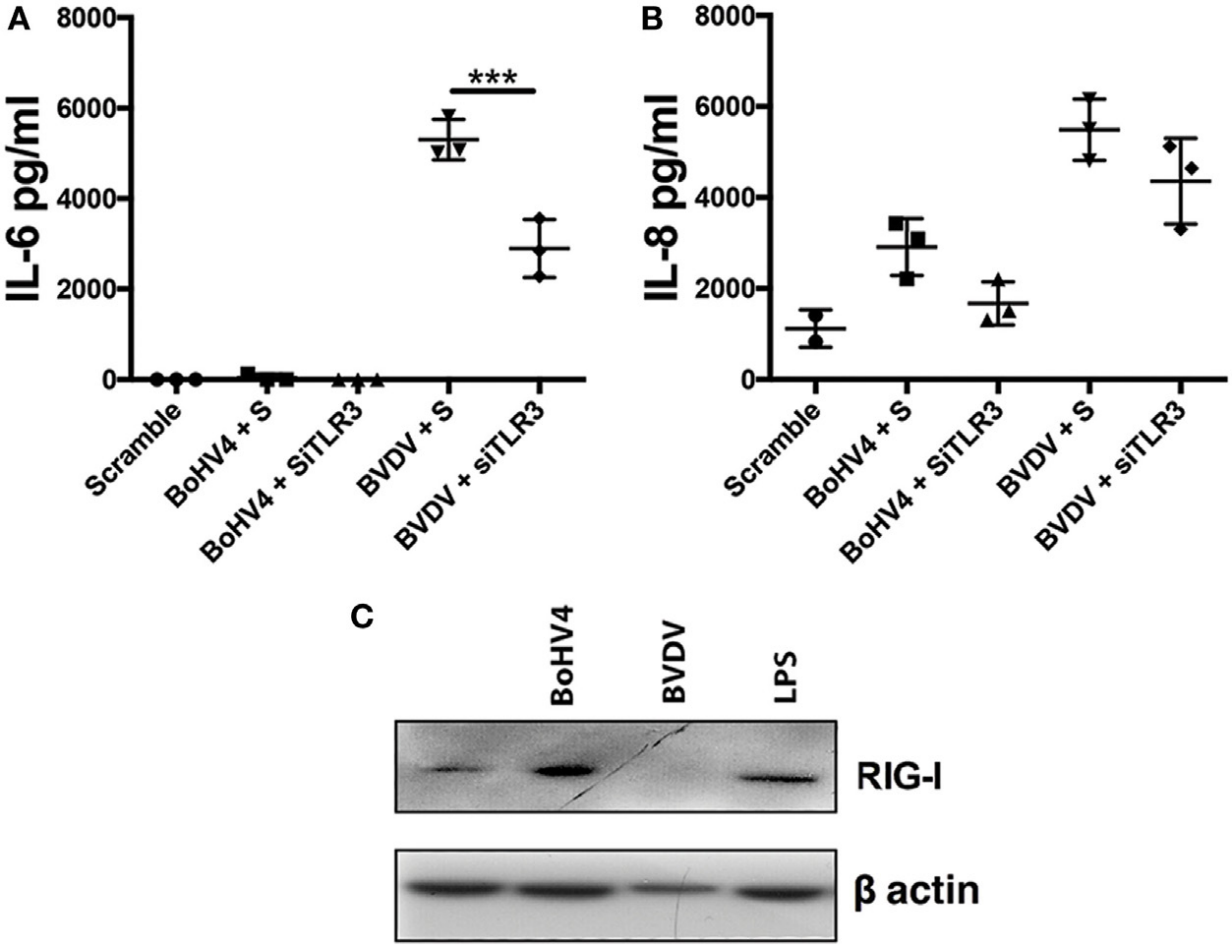
Toll-like receptor (TLR)-3 moderate’s virus induced inflammatory mediator production in endometrial stromal cells. Stromal cells **(A,B)** were cultured for 72 h in medium plus scrambled short interfering RNA (siRNA) (S) or media containing bovine herpesvirus-4 (BoHV-4) or bovine viral diarrhea virus (BVDV) [at 1 multiplicity of infection (m.o.i.)]. In each independent set of experiments, cells received vehicle plus scrambled siRNA control (scramble), vehicle plus siRNA targeting TLR3 (siTLR3) 18 h before 72 h virus treatment. Concentrations of IL-6 **(A)** or IL-8 **(B)** in supernatants were measured by enzyme-linked immunosorbent assay. Data represent three independent animal experiments. Data are presented as mean + SEM, and analyzed by two-way analysis of variance, using the *post hoc* Dunnett’s multiple comparison test to compare siRNA plus virus to S plus virus, ***P < 0.001. Stromal cells **(C)** were cultured for 96 h in medium containing lipopolysaccharide (1 µg/ml), BoHV-4, or BVDV (at 1 m.o.i.). Total cell proteins were extracted, and analyzed by immunoblotting for retinoic acid-inducible gene I and to β-actin.

All viruses, except negative-strand RNA viruses, generate dsRNA during genome replication, a TLR3 ligand (23). As TLR3 was important for IL-6 production in response to transfected Poly(I:C), we next investigated the influence of TLR3 on inflammatory mediator production in response to viable BoHV-4 or BVDV (Figures 7A,B). Accordingly, depletion of TLR3 using siRNA resulted in reduced IL-6 production in response to BVDV (Figure 7A). Finally, to investigate whether BoHV-4 or BVDV induce increased expression of RIG-I in stromal cells, stromal cells were exposed to viable virus for 96 h. BoHV-4 induced an increase in RIG-I expression, whereas RIG-I expression was reduced in BVDV treated cells (Figure 7C).

## Discussion

Bovine herpesvirus-4 and BVDV cause uterine disease in cattle, often resulting in reduced fertility, or abortion of the fetus, respectively (11). Countering viral infections requires coordination of the innate immune system by host cells, including pathways initiated by PRRs and the appropriate production of cytokines, chemokines, and IFNs. In this study, we demonstrate that BVDV induced cytokine and chemokine production in EVOCs and endometrial epithelial cells, and IL-6 production in stromal cells. Whereas, BoHV-4 did not induce accumulation of any of the tested inflammatory cytokines from EVOCs. In epithelial cells, IL-6 was reduced and IL-8 increased upon treatment with BoHV-4. BoHV-4 increased stromal IL-8 production, but there was no observed change in IL-6. These data indicate that endometrial cells initiate differing innate immune signaling pathways, dependent on the specific viral challenge.

A previous study showed that endometrial cells co-treated with LPS and BVDV had altered expression of genes associated with the innate immune response to viruses, including *DDX58* (RIG-I) (8). Our data show that RIG-I is barely detectable in untreated primary epithelial or stromal cells. However, transfection of Poly(I:C), a mimetic of viral dsRNA, induced RIG-I protein expression in a time- and concentration-dependent manner in endometrial stromal, but not epithelial cells. Treatment of stromal cells with viable BoHV-4 also induced increased RIG-I levels. Although BoHV-4 is a DNA virus, all viruses, except negative-stranded RNA viruses, have a dsRNA stage in their lifecycle (23).

Infection with RNA viruses induces cytokine and chemokine production in a TLR-dependent manner (41, 42). Because RNA is a universal viral molecular pattern, TLR3 has been assumed to have a central role in the host response to most viruses (43). For example, infection with West Nile virus, an ssRNA virus, initiates an inflammatory response through TLR3, as *Tlr3^−/−^* mice are more resistant to infection with the virus (44). Bovine endometrial cells increase gene expression of TLR3, complement, and chemotactic and TRIM factors in response to BVDV (8). However, despite BVDV inducing increases in inflammatory cytokine production in EVOCs, epithelial, and stromal cells, in our study increased RIG-I protein was not evident in BVDV treated stromal cells. This may indicate that endometrial cells use pathways other than RIG-I, downstream of TLR3, to initiate inflammatory cytokine production in response to BVDV.

Unlike signaling pathways initiated by all other TLRs, TLR3 does not recruit the adaptor MyD88, but solely depends on the adaptor TRIF. The TRIF signaling pathway leads to the activation of NF-κB and IRF3, key transcription factors, with roles in innate immunity (45). An essential step for IRF3 activation is mediated by the recruitment of TBK1 to TRIF. Our data demonstrate that Poly(I:C) induces phosphorylation of NF-κB and TBK1, suggesting a functional TLR3 pathway in endometrial stromal cells. This is further evidenced by using siRNA, as depletion of *TLR3* or *IRF3* resulted in reduced RIG-I, indicating an importance for this pathway in RIG-I upregulation. Furthermore, depletion of *TLR3* reduced Poly(I:C) induced IL-6 and IL-8 accumulation, and BVDV induced IL-6 in stromal cell supernatants, demonstrating the importance of this pathway in cytokine and chemokine production in response to virus. Interestingly, although IRF3 was important for RIG-I expression, depletion of *IRF3* did not affect IL-6 or IL-8 production. However, we cannot exclude the contribution of other DNA sensors in endometrial stromal cell signaling. Thus, multiple pathways are probably involved in controlling early viral replication in stromal cells *in vivo*.

Our data does not establish a direct link between BoHV-4, dsRNA, and RIG-I expression. For example, in the first few hours of stromal infection with cytomegalovirus, a herpesvirus, TLR independent IFN-I responses were dependent on cGAS, STING, and IRF3 signaling (46). Thus, multiple pathways are involved in controlling early viral replication in stromal cells *in vivo* and there is a possibility that other viral PAMPs or components may be capable of inducing RIG-I expression through other PRRs. For instance, unmethylated viral CpG-DNA and viral ssRNA stimulate TLR9- and TLR7-dependent signaling pathways, respectively (30, 40). However, our data indicate that transfected dsDNA or ssRNA did not induce increased RIG-I in endometrial cells. DNA is also detected intracellularly *via* AIM2, which initiates the formation of the inflammasome complex, to orchestrate mature IL-1β release from cells (20). Our results show a marginal increase in IL-1β in response to the DNA virus BoHV-4, but a significant increase in IL-1β from BVDV, an RNA virus. As well as RIG-I, the RLR family includes the cytoplasmic sensor MDA-5, which is widely expressed in many cell types (47). In a previous study, expression of several IFN-inducible genes, including *IFIH1*, were significantly increased in cows suffering a severe negative energy balance status (48). Whether this is a result of increased viral load in these cows is unclear. Unfortunately, in the present study, we were unable to find a suitable bovine specific MDA-5 antibody to use, and ELISAs specific for bovine type I IFNs are unavailable.

Replication deficient BoHV-4 viruses are still able to infect endometrial epithelial and stromal cells, but the virus does not induce the production of proinflammatory cytokines or chemokines. This may suggest that the virus is only detected once viral replication occurs. Only then, once dsRNA is produced, can the cells initiate an innate immune response *via* TLR3 and/or RIG-I. As RIG-I is a cytosolic receptor, stroma may be particularly vulnerable to BoHV-4 CPE as it takes up to 96 h for increased RIG-I protein to become evident. Other factors may also play a part in the pathogenesis of viral infection, other than PRRs. For example, studies show that the BoHV-4 *IE2* (ORF50/*Rta*) gene transactivates the *CXCL8* (IL8) gene promoter, either directly or indirectly during BoHV-4 infection (49). Therefore, IL-8 production may actually be of benefit to the virus.

In conclusion, we report that bovine endometrial cells are capable of detecting and responding to virus, and their PAMPs, through TLR3 and RIG-I. The relative contribution of PRRs in the innate defense of endometrial cells to viruses requires further study to delineate the specific roles each contributes. However, from our data it would appear that the response to viruses and their ligands requires RIG-I in a coordinated response orchestrated by TLR3, at least in regard to detection of viral dsRNA.

## Ethics Statement

Uteri with no gross evidence of genital disease or microbial infection were collected from cattle processed as part of the normal work of an abattoir.

## Author Contributions

JC, IMS, VL, and GD designed the study. JC and IMS wrote the manuscript. LC, CB, SJ, AR, and JC carried out all the experimental work.

## Conflict of Interest Statement

The authors declare that the research was conducted in the absence of any commercial or financial relationships that could be construed as a potential conflict of interest.
