# Supplementary material for: Coordinated Role of Toll-Like Receptor-3 and Retinoic Acid-Inducible Gene-I in the Innate Response of Bovine Endometrial Cells to Virus
Source: Front Immunol. 2017 Aug 23;8:996. doi: 10.3389/fimmu.2017.00996 (PMC5572515; doi:10.3389/fimmu.2017.00996)
Supplement: Supplementary file 1 [file Table_1.DOCX]

Supplemental Table 1. siRNA sequences.

| Gene  Symbol | Gene Name | Duplex Sequence | Genbank  Accession number |
| --- | --- | --- | --- |
| *TLR3* | Toll like receptor 3 | Sense: 5’ GCU GAA GGG UCU UGA GAA AUU 3’  Antisense: 5’ UUU CUC AAG ACC CUU CAG CUU 3’ | NM_001008664.1 |
| *IRF3* | Interferon regulatory factor 3 | Sense: 5’ GGA AGG AAG UGU UGC GUU UUU 3’  Antisense: 5’ AAA CGC AAC ACU UCC UUC CUU 3’ | NM_001029845.3 |
| *TRAF3* | TNF receptor associated factor 3 | Sense: 5’ GGA CAA ACC AGC AGA UCA AUU 3’  Antisense: 5’ UUG AUC UGC UGG UUU GUC CUU 3’ | NM_001205586.1 |
| *MYD88*  *(ref. 3)* | Myeloid differentiation  primary response gene (88) | Sense: 5’ ACG AAU ACC UGC AAA GCA AUU 3’  Antisense: 5’ UUG CUU UGC AGG UAU UCG UUU 3’ | NM_001014382 |
